# Relative Susceptibilities of ABO Blood Groups to *Plasmodium falciparum* Malaria in Ghana

**DOI:** 10.1155/2016/5368793

**Published:** 2016-02-15

**Authors:** Richmond Afoakwah, Edmond Aubyn, James Prah, Ekene Kwabena Nwaefuna, Johnson N. Boampong

**Affiliations:** ^1^Department of Biomedical and Forensic Sciences, University of Cape Coast, Cape Coast, Ghana; ^2^University Hospital, University of Cape Coast, Cape Coast, Ghana; ^3^Vector Genetics Laboratory, Biotechnology and Nuclear Agriculture Research Institute, Atomic Energy Commission, Accra, Ghana

## Abstract

The clinical outcome of falciparum malaria in endemic areas is influenced by erythrocyte polymorphisms including the ABO blood groups. Studies have reported association of ABO blood group to resistance, susceptibility, and severity of* P. falciparum *malaria infection. Individuals with blood group “A” have been found to be highly susceptible to falciparum malaria whereas blood group “O” is said to confer protection against complicated cases. We analyzed samples from 293 young children less than six years old with malaria in the Korle-Bu Teaching Hospital in Accra, Ghana. It was observed that group O was present in about 16.1% of complicated cases weighed against 40.9% of uncomplicated controls. Individuals with complicated malaria were about twice likely to be of blood groups A and B compared to group O (A versus O, OR = 1.90, 95% CI = 1.59–2.26, *P* < 0.0001; B versus O, OR = 1.82. 95% CI = 1.57–2.23, *P* < 0.0001). Blood group O participants with complicated diseases had low parasitaemia compared to the other blood groups (*P* < 0.0001). This may give blood group O individuals a survival advantage over the other groups in complicated malaria as suggested. Participants with complicated falciparum malaria were generally anaemic and younger than those with uncomplicated disease.

## 1. Introduction

*Plasmodium falciparum *malaria is a known cause of morbidity and mortality especially in children of Sub-Saharan Africa [1]. The clinical outcome of falciparum malaria in endemic areas is, among other factors, associated with erythrocyte polymorphisms [2, 3] including the ABO blood groups. ABO blood group refers to a system of carbohydrate antigens expressed on human erythrocytes [4] and other human cells. The “A” and “B” antigens on erythrocytes are trisaccharides [5]. All erythrocytes possess an “H” disaccharide on their surfaces (except the rare Bombay phenotype, which has no ABO antigens) [4]. Individuals with blood groups “A” and “B” have the “A” and “B” antigens, respectively, together with the “H” antigen. Blood group “AB” individuals have both “A” and “B” antigens together with the “H.” Blood group “O” individuals, however, have neither “A” nor “B” antigens but “H.”

Numerous associations have been reported between the ABO blood group system and some disease conditions such as skin cancer [6], schistosomiasis [7], onchocerciasis [8], and HIV infection [9]. There are also reports on association of ABO blood group with susceptibility, resistance, and severity of* P. falciparum *malaria infection [10–15]. Individuals with blood group “A” have been found to be highly susceptible to falciparum malaria whereas blood group “O” is said to confer protection against complicated cases [11–13]. Low parasitaemia and uncomplicated* P. falciparum *malaria cases among blood group “O” individuals have been observed [11, 14, 15].

These differences in susceptibility and severity of* P. falciparum *malaria infection among the “A,” “B,” “AB,” and “O” blood groups have been attributed to rosetting of parasitized erythrocytes and cytoadherence [16–19]. Rosetting contributes to the pathogenesis of severe malaria by obstructing microvascular blood flow [20]. Studies have shown that rosetting is reduced in blood group “O” erythrocytes compared with the non-O blood groups (A, B, and AB) in* P. falciparum *laboratory [21, 22] and field isolates [19, 23]. Rosettes may form in blood group “O” cells but these rosettes are smaller and unstable compared to rosettes formed in non-O blood groups [21, 24, 25]. It is, thus, presumed that blood group “O” may be a protective factor against severe malaria [25].

Some studies have also reported the absence of significant association between ABO blood groups and* P. falciparum *malaria [7, 8, 26–30], so that the relationship between ABO blood group and malaria has not been clearly defined [4]. This study aimed to confirm the association, or otherwise, between blood groups and complicated falciparum malaria.

## 2. Methods

The study was conducted from January to April 2010, at the outpatient department of the Korle-Bu Teaching Hospital in Accra, Ghana. The study was reviewed and cleared by the University of Cape Coast Ethics Review Board. The study was explained to parents/guardians of prospective participants and informed consent was sought from them. A participant was eligible for inclusion into the study if his/her age at the next birthday was 5 years or less and had been diagnosed to have falciparum malaria.

Five milliliters (5 mL) of blood sample was collected from each patient into EDTA tubes by trained and licensed medical laboratory technologists. Sterile techniques and disposable, single use materials were used at all times.

The hemoglobin level of each participant was determined using a Hematology Analyzer (Abbott Cell-Dyn CD-1800). Giemsa-stained thick and thin blood films were prepared for each sample collected, from which parasite density and species identification were, respectively, determined. Species confirmation of the malaria parasite was done with first response PfHRP-II malaria rapid-diagnostic (RDT) kit. Cerebral malaria (involving drowsiness, impaired consciousness, recurrent convulsion, and/or unrousable coma) and hyperparasitaemia (with parasite density ≥6%) were considered the primary criteria for defining complicated malaria.

ABO blood groups were typed by the agglutination method using commercial antisera (Span Diagnostics Ltd., India).

Data obtained were analyzed with Minitab Statistical Software version 15. Results were presented as mean ± standard deviation or percentages where appropriate. Mean values were compared using either Student's *t*-test or One-Way ANOVA. In addition, odds ratios and 95% confidence interval were determined. In all the analyses, values were considered to be significant when *P* ≤ 0.05.

## 3. Results

A total of 293 young children were recruited for the study. Of these, 112 (41.3%) had complicated falciparum malaria, whereas 181 (58.7%) had uncomplicated falciparum cases. The characteristics of the participants are summarized in Table 1.

Respondents in the complicated group were younger and anaemic and experienced higher parasitaemia compared with their counterparts in the uncomplicated group (*P* < 0.05; Table 1). Of the 293 participants, 92 (38.5%), 74 (30.96%), 72 (30.13%), and 55 (23.01%) had blood groups O, B, A, and AB, respectively. Blood groups of the participants influenced the development of complicated falciparum malaria as shown in Table 2.

The chance of developing complicated falciparum malaria was least in blood group O compared to blood groups A, B, and AB (Table 3).

## 4. Discussion

In high transmission areas like Ghana, by age six children have survived several episodes of malaria and hence developed some immunity to the infection [30–32]. Increasing age has, thus, been linked to lower parasite densities and consequently less complicated disease [33] making older children and adults in high transmission areas have less readily detectable infections [34]. We, therefore, chose to recruit children younger than six years of age to remove the confounding effect age-dependent immunity to malaria would have on the study. Indeed our data supported this notion since participants who had complicated falciparum malaria were significantly younger than those who had uncomplicated disease.

Adherence of* P. falciparum *parasitized erythrocytes to the endothelia of blood vessels is key to the pathogenesis of complicated disease [2, 31]. Antigens of blood groups A and B have been suggested to play important roles in cytoadherence [32]. Due to the absence of A and B antigens on the surface of blood group O erythrocytes, cytoadherence, and hence rosetting and sequestration, is reduced in individuals with blood group O [17]. It has been observed that blood group O individuals are less likely to suffer from complicated falciparum malaria [11, 15, 25, 35, 36].

In this study, we observed that only 18 (16.1%) of the 112 participants with complicated disease had blood group O, whereas as much as 74 (40.9%) of the 181 participants with uncomplicated disease had blood group O. This observation gives credence to results of previous studies [11, 15, 25, 35, 36]. Individuals with complicated malaria were about twice likely to be of blood group A or B compared to group O.

The low parasitaemia observed in this study, together with reduced rosetting and cytoadherence observed by others [17, 27–29], may give the blood group O individuals suffering from falciparum malaria a good prognosis compared with those with other blood groups. In addition, this observation supports the view that blood group O individuals may have a survival advantage over non-O individuals notably in complicated cases of falciparum malaria [27]. However, the comparable levels of hemoglobin observed among individuals in the various blood groups in both complicated and uncomplicated cases suggest increased rate of destruction of red blood cells (RBCs) in blood group “O” individuals compared to the others. The significance of anaemia in the pathogenesis of complicated falciparum malaria has been documented [16, 19]. Destruction of both parasitized and nonparasitized erythrocytes as well as rosetting and sequestration of parasitized erythrocytes has been cited to be the major cause of severe anaemia in complicated falciparum malaria [16, 19]. Our data in the present study appear to suggest that the RBCs of blood group “O” individuals were more susceptible to falciparum-induced hemolysis than the RBCs of individuals of other blood groups. Thus, the apparent protection offered by blood group “O” may be lost at relatively higher levels of parasitaemia. This observation is important for the malaria control effort in Ghana.

## 5. Conclusion

Ghanaian children with blood group O may have some protection against complicated falciparum malaria and may possess a survival advantage over their counterparts with other blood groups. However, this protection may be lost at high parasitaemia due to enhanced RBC destruction. Younger children are also more prone to developing complicated falciparum malaria than older ones.

## Figures and Tables

**Table 1:** Characteristics of participants.

Category	Age (years)	Hb (g/dL)	Parasite density (% of parasitized RBCs)
Complicated cases	2.384 (±1.239)	9.579 (±0.900)	10.542 (±2.704)
Uncomplicated controls	2.796 (±1.315)	12.338 (±1.278)	7.596 (±2.040)
*P* value	*0.007*	*<0.0001*	*<0.0001*

**Table 2:** Blood group distribution in complicated cases and uncomplicated controls.

Category	*N*	Parasite density (% of parasitized RBCs)	Hb (g/dL)
Complicated cases			
A	32	11.906 (±2.487)	9.705 (±0.862)
AB	26	9.088 (±1.583)	9.430 (±0.948)
B	36	11.400 (±2.965)	9.536 (±0.883)
O	18	8.497 (±1.402)	9.656 (±0.965)
*P* value (ANOVA)		*<0.0001*	*0.676*
Uncomplicated controls			
A	40	8.567 (±1.943)	12.358 (±1.262)
AB	29	7.610 (±2.123)	12.537 (±1.526)
B	38	8.605 (±1.886)	12.237 (±1.084)
O	74	6.546 (±1.596)	12.300 (±1.291)
*P* value (ANOVA)		*<0.0001*	*0.800*

**Table 3:** Effect of blood group on developing complicated falciparum malaria.

Blood groups	Odds ratio	*P* value	95% CI	
			Lower	Upper
B versus O	1.87	<0.0001	1.57	2.23
AB versus O	1.40	<0.0001	1.18	1.66
A versus O	1.90	<0.0001	1.59	2.26
